# Simultaneous ^18^F-FDG-PET/MRI for the detection of periprosthetic joint infections after knee or hip arthroplasty: a prospective feasibility study

**DOI:** 10.1007/s00264-022-05445-7

**Published:** 2022-05-30

**Authors:** Jeanette Henkelmann, Ralf Henkelmann, Timm Denecke, Dirk Zajonz, Andreas Roth, Osama Sabri, Sandra Purz

**Affiliations:** 1 Department for Diagnostic and Interventional Radiology, University Hospital Leipzig, Liebigstraße 20, 04103 Leipzig, Germany; 2 Department for Orthopaedics, Trauma and Plastic Surgery, University Hospital Leipzig, Leipzig, Germany; 3 Clinic for Orthopaedics, Trauma and Reconstructive Surgery, Zeißigwald Hospital Bethanien Chemnitz, Chemnitz, Germany; 4 Department of Nuclear Medicine, University Hospital Leipzig, Leipzig, Germany

**Keywords:** Periprosthetic infections, Positron emission tomography, Magnetic resonance imaging, Hip and knee arthroplasty

## Abstract

**Purpose:**

This study investigated the diagnostic value of simultaneous ^18^F-fluordeoxyglucose positron emission tomography/magnetic resonance imaging (PET/MRI) in suspected periprosthetic joint infection (PJI) of the hip and knee.

**Methods:**

Sixteen prostheses from 13 patients with suspected PJI were prospectively examined using PET/MRI. Image datasets were evaluated in consensus by a radiologist and a nuclear physician for the overall diagnosis of ‘PJI’ (yes/no) and its anatomical involvement, such as the periprosthetic bone margin, bone marrow, and soft tissue. The imaging results were compared with the reference standard obtained from surgical or biopsy specimens and subjected to statistical analysis.

**Results:**

Using the reference standard, ten out of the 13 prostheses (ten hips, threes knees) were diagnosed with PJI. Using PET/MRI, every patient with PJI was correctly diagnosed (sensitivity, 100%; specificity, 100%). Considering the anatomical regions, the sensitivity and specificity were 57% and 50% in the periprosthetic bone margin, 75% and 33% in the bone marrow, and 100% and 100% in the soft tissue.

**Conclusion:**

PET/MRI can be reliably used for the diagnosis of PJI. However, assessment of the periprosthetic bone remains difficult due to the presence of artefacts. Thus, currently, this modality is unlikely to be recommended in clinical practice.

**Supplementary Information:**

The online version contains supplementary material available at 10.1007/s00264-022-05445-7.

## Introduction

Primary hip and knee arthroplasty is one of the five most frequently performed procedures annually [1]. Complication rates for elective hip or knee arthroplasty range from 0.5 to 10%, with periprosthetic joint infection (PJI, 14.5–25.1%) being the third most common complication after aseptic loosening and dislocation, occurring with a latency of less than three months (early) to more than 24 months (late) [2–6].

Accurate and early diagnosis of PJI is crucial to reduce morbidity [7, 8]. The distinction between aseptic loosening and bacterial infection is important, as it affects the surgical method [9]. PJI differs from simple bone and joint infections as a complex entity. The implant colonised with microorganisms becomes a permanent reservoir, which makes successful diagnosis and treatment difficult [10, 11]. The proof of PJI is through microbiological detection of pathogens by tissue sampling or intraoperative smears. This is preceded by clinical examination, blood tests, and radiological and nuclear imaging techniques.

In conventional radiography, which is usually the modality used first, typical signs of PJI can be indicated by periprosthetic osteolysis, loosening margins, or periarticular calcifications. However, these signs are late indicators. Cross-sectional imaging using computed tomography (CT) or magnetic resonance imaging (MRI) can be affected by artefacts due to metal implants. In addition, post-operative changes are sometimes difficult to distinguish from signs of PJI. Metabolic changes usually precede morphologic findings, and functional imaging modalities can usually reveal pathological findings earlier than conventional imaging. Scintigraphic techniques, such as antigranulocyte scintigraphy, have a sensitivity of up to 95% [12]. With a significantly better spatial resolution, ^18^F-fluorodeoxyglucose positron emission tomography (^18^F-FDG PET) is applied, especially for the exclusion of PJI [13, 14]. Nevertheless, MRI offers the highest spatial and tissue resolution in this regard and visualises periprosthetic bone and soft tissues [15]. Technical innovations in recent years have established metal artefact reducing sequences and allow assessment of post-operative complications after arthroplasty [16, 17].

Hybrid imaging of combined and simultaneous MRI and PET potentially detects early changes in infected tissue and morphologically images the precise extent of PJI (spread in bone or soft tissue). Simultaneous ^18^F-FDG-PET/MRI has been a powerful diagnostic tool for spinal infections [18]; however, its value in PJI has not been systematically studied [19].

This monocentric, prospective exploratory pilot study aimed to evaluate the clinical applicability of simultaneous acquisition of ^18^F-FDG-PET/MRI in the diagnosis of PJI and to assess its sensitivity and specificity.

## Materials and methods

### Patient population

This single-centre study prospectively enrolled 13 patients with clinically inconclusive suspected PJI after initial prosthesis implantation between December 2018 and December 2019. Informed written consent was obtained from each patient prior to enrolment. The trial was registered in the German Registry of Clinical Trials (DRKS00021211). The study was approved by the local ethics committee (335–17-ek) and performed in accordance with the Declaration of Helsinki. This study focuses on uncertain suspected cases of PJI. In patients with suspected PJI and multiple arthroplasties (knee or hip), all arthroplasties were considered potentially infected and included in the study. The following inclusion criteria were defined: patients with clinically inconclusive suspected PJI after hip or knee arthroplasty, age > 18 years and MRI compatibility of hip or knee arthroplasty. Patients with evident PJI, for example with definite joint empyema and/or remarkable radiographic signs of loosening, were excluded in this study. Further exclusion criteria were applied: pregnancy or breastfeeding, decompensated diabetes mellitus or fasting blood glucose values > 12 mmol/l on the day of examination, general MRI contraindications (e.g. non-MRI compatible implants, claustrophobia), contrast agent intolerance, incomplete image datasets (e.g. motion artefacts), and renal insufficiency (glomerular filtration rate < 30 ml/min). Possible previous interventions (e.g. biopsy and arthroscopy) in the examination area had to be at least 8 weeks before the examination day.

The primary outcomes were the clinical applicability and sensitivity and specificity of PJI by simultaneous acquisition of ^18^F-FDG PET/MRI. The secondary outcome was the extent of infection in the periprosthetic bone margin, bone marrow of the femoral or tibial shaft, and the soft tissue using the aforementioned imaging modalities.

### Simultaneous ^18^F-FDG PET/MRI

The combined PET/MRI system (mMR-Biograph®, Siemens Healthcare, Erlangen, Germany) used in this study combines a 3 T MRI with an integrated PET scanner. Whole-body sequential PET/MRI scanning was performed from the lower thigh to the skull with a five minute acquisition time per bed position. Image acquisition commenced on an average of 75 (range 60–105) min after intravenous administration of ^18^F-FDG at a dose of 4 MBq/kg after a fasting period of at least six hours. Attenuation correction of the PET data was performed with a four-tissue (fat, soft-tissue, air, and background) model attenuation map obtained from a Dixon–volume-interpolated breath-hold examination MR sequence.

MR images of the hip or knee were obtained in the neutral lying position using a 16-channel body array coil. The examination protocol comprised a coronal turbo inversion recovery magnitude sequence, coronal T1-weighted (T1W) sequence without fat suppression prior to and after intravenous administration of a gadolinium-based contrast medium, sagittal T1W sequence, and axial T2W sequence without fat suppression (Table 1). All sequences were acquired with high bandwidth parameters and view angle tilting to reduce metal artefacts from prosthesis components. For contrast-enhanced MRI, a single gadobutrol dose of 0.1 mmol/kg, at a rate of 3 mL/s and flushed with 10 mL of saline, was administered.

**Table 1:** Scanning parameters. TE, echo time; TR, repetition time; FOV, field of view

Scanning parameters	Coronal TIRM	Coronal T1W sequence	Axial T1W sequence	Axial T2W sequence	Coronal T1 post-contrast
TR (ms)	8000	750	800	4000	750
TR (ms)	22	8.1	8.1	73	8.1
Slice thickness (mm)	2.5	2.5	2.5	2.5	2.5
FOV (mm × mm)	400 × 400	400 × 400	400 × 400	400 × 400	400 × 400
Bandwidth (Hz/px)	504	797	797	507	797
Flip angle (°)	146	135	136	141	135
Acquisition time (s)	3:30	3:23	3:29	2:42	3:23

### Image interpretation

An interdisciplinary analysis of the PET/MRI datasets was performed together with a nuclear medicine specialist with ten years experience and a musculoskeletal board-certified radiologist with six years experience. Both readers only received information on the clinical suspicion of PJI and the area of the patients’ pain. Image analysis was blinded for both readers to laboratory parameters and histopathology/microbiologic results. The PET/MRI image studies were evaluated in combination simultaneously by both readers, taking into account the following findings: PET findings were considered positive for PJI if noticeably increased diffuse ^18^F-FDG uptake was detected periprosthetically (especially at the bone–prosthesis interface, which suggests infection) or in the surrounding tissue and negative if there was no diffuse uptake in this region. The mean and maximum standardised uptake values (SUVmean and SUVmax) were determined by placing a volume of interest around the respective region (SUVmax threshold, 40%). PJI-suspicious MRI findings included T2W-hyperintense signal alterations and contrast enhancement at the metal–bone interface and the periosteum, significant alterations in the surrounding bone marrow and surrounding soft tissue, muscle oedema, and presence of fluid collections or an abscess. The diagnosis were dichotomised into ‘PJI’ and ‘no PJI’. The evaluation was performed for the overall diagnosis of ‘PJI’ and separately for each anatomical region: periprosthetic bone margin, bone marrow of the femoral or tibial shaft, and the soft tissue.

### Reference standards

The imaging results were compared with the results of histopathological and microbiological analyses of surgical or biopsy specimens as reference standards following a major criterion of PJI according to Parvici et al. [20]. Depending on the extent of the surgical procedure, samples of the implantation sonication, soft tissues, or bone marrow may be available for evaluation.

### Statistical analysis

If not otherwise stated, for quantitative variables, the descriptive normally distributed data are given as mean ± standard deviation and the non-normally distributed as median and range.

Sensitivity, specificity, positive predictive value, and negative predictive value were calculated and reported as a percentage. Statistical analysis was performed using commercial software (IBM SPSS Statistics for Windows, Version 24.0. IBM Corp. Armonk, NY, USA).

## Results

Thirteen (seven men, six women; median age, 71.5 [range, 52–85] years) patients underwent simultaneous ^18^F-FDG-PET/MRI examination. The mean time between implantation and PET/MRI was 5.8 (min–max, 0.5–7.8) years. Ten patients were included in the final analysis (Table 2). Three patients were excluded because their image files were not analysable due to incomplete image acquisition (restlessness and excessive movement artefacts).

**Table 2:** Patient characteristics. *ASA*, Physical Status Classification of the American Society of Anesthesiologists; *CCI*, Charlson Comorbidity Index

Patient no	Sex	Age	CCI	ASA	Prosthesis localisation		Prosthesis no
1	F	80	7	3	Hip	Right	1
2	M	77	8	3	Hip bilateral	Right	2
						Left	3
3	M	55	4	2	Hip	Left	4
4	M	53	2	1	Hip	Right	5
5	F	70	7	3	Hip	Left	6
6	F	74	4	2	Knee	Right	7
7	F	75	5	2	Knee	Left	8
8	M	73	4	2	Knee bilateral	Right	9
						Left	10
9	F	54	4	2	Hip	Right	11
10	M	58	4	3	Hip bilateral	Right	12
						Left	13

Thirteen prostheses were analysed, as three patients had bilateral prosthesis implants. Joint prosthesis complaints were reported (median duration, 4 ± 2 [range, 1–7] years). PET/MRI was performed for a mean of six (range, 3–15) days before the reference standard.

**Fig. 1:**
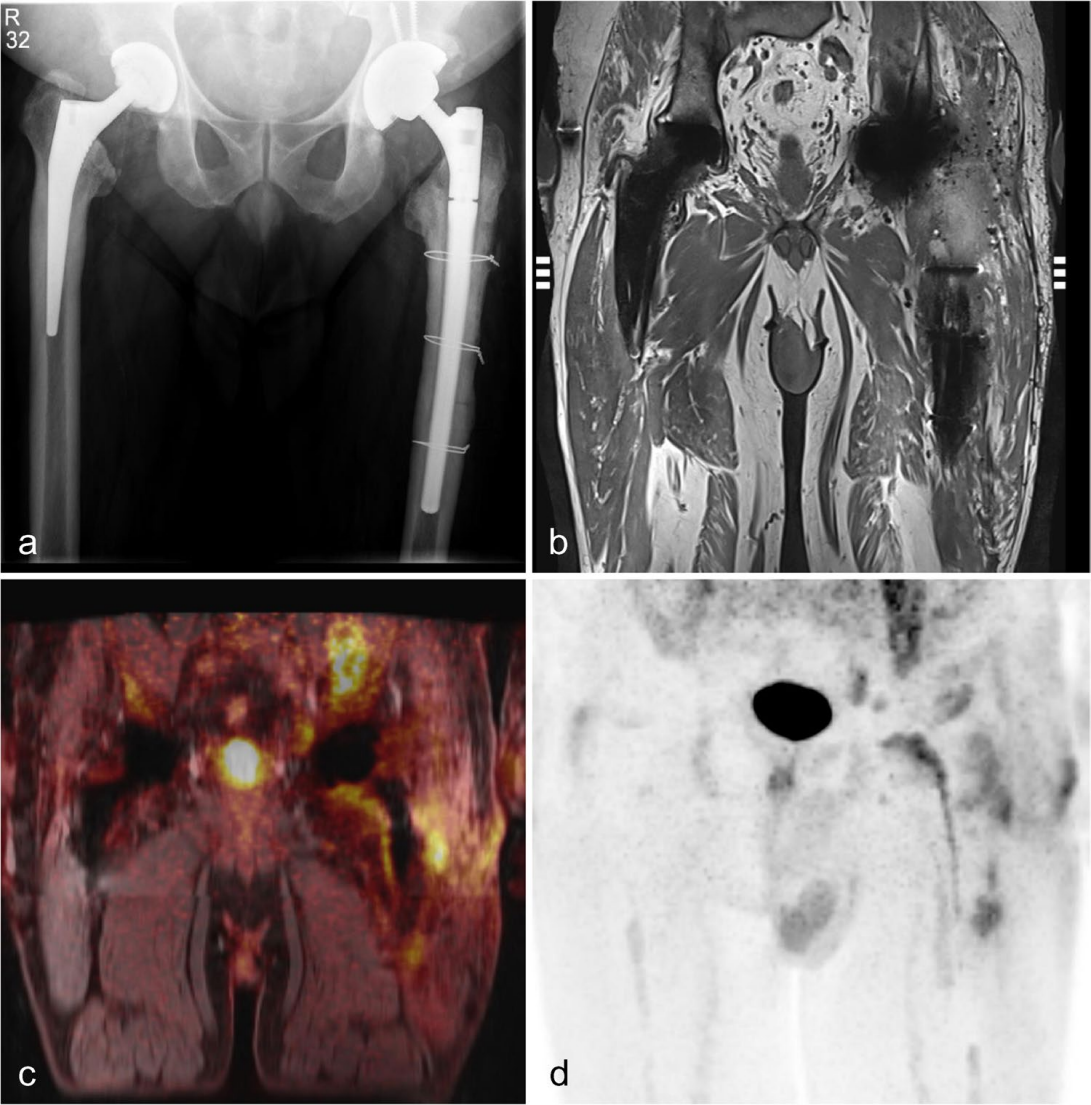
**A** X-ray of a 55-year-old man with bilateral hip prostheses and left-sided cerclage. **B** Coronal T1-weighted magnetic resonance imaging (MRI) shows slight metal artefacts in both prostheses and left-sided fluid collections around the neck of the prosthesis. Fused.^18^F-FDG-PET/MRI (**C**) and the maximum intensity projection image (**D**) show significant elevated tracer uptake around the prostheses neck, shaft, and soft tissue. In contrast, the left non-infected hip prosthesis reveals a normal tracer pattern. The findings were confirmed by reference standard

**Fig. 2:**
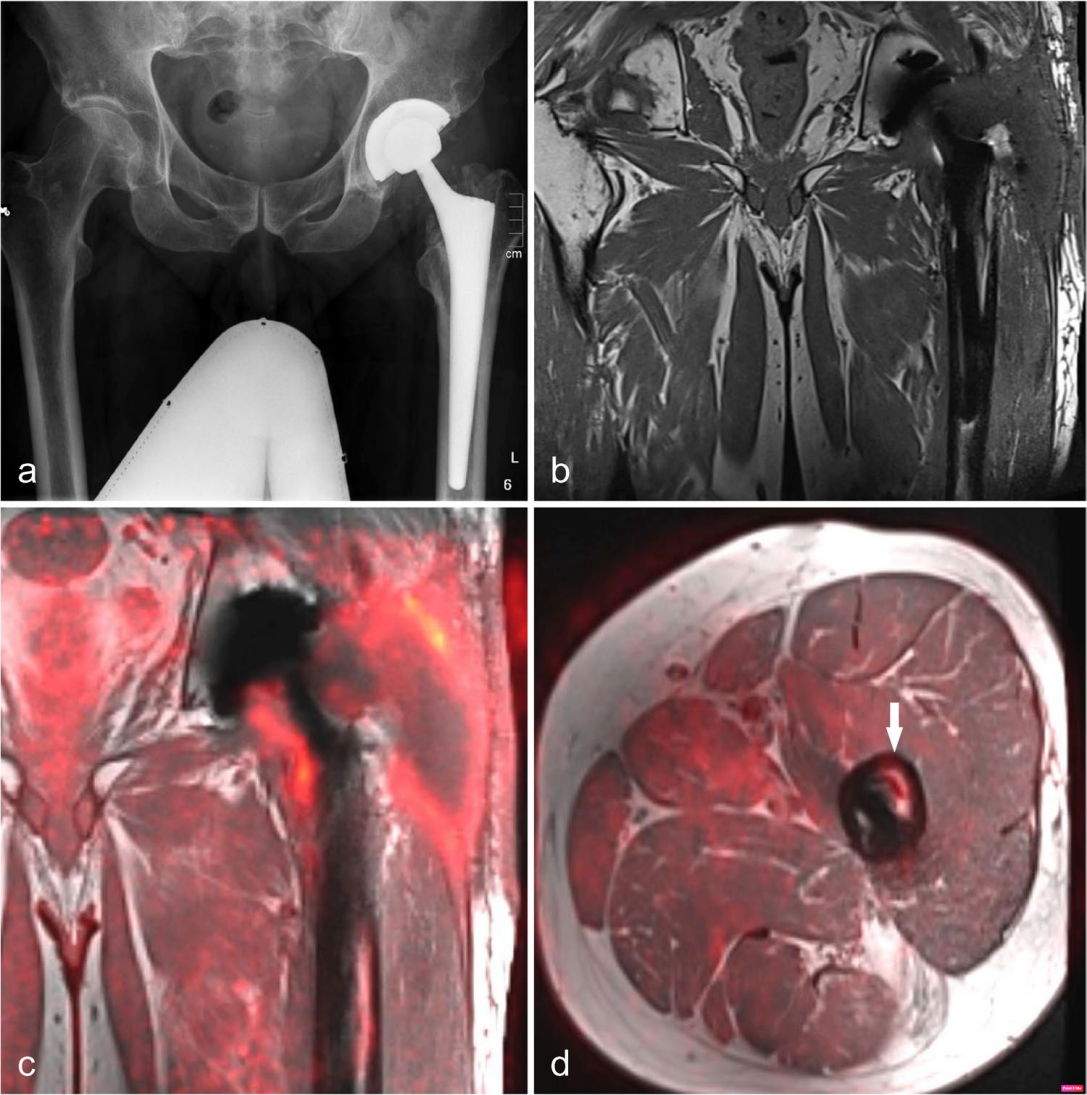
**A** X-ray of a 54-year-old man with left-sided hip prosthesis. **B** Coronal T1-weighted magnetic resonance imaging (MRI) shows distinct subfacial fluid collections extending around the prostheses neck. Fused.^18^F-FDG-PET/MRI (**C** + **D**) shows an elevated tracer uptake around the prosthesis neck, shaft, and soft tissue. The increased uptake in the periprosthetic bone marrow of the femur (arrow) was confirmed as inflammatory involvement by prosthesis revision and sonication

According to the reference standard, ten of the 13 prostheses were diagnosed with PJI. In three patients with bilateral implants, PJI of the nonpainful prosthesis was ruled out by joint puncture. In ten prostheses, there was an indication for surgical revision, and PJI was confirmed. Thus, ten implant sonication samples, ten soft tissue samples, and seven samples of periprosthetic bone marrow were analysed. Figure 1 shows a patient with bilaterally implanted hip prosthesis and left-sided increased ^18^F-FDG uptake surrounding the prosthesis on histologically proven PJI. In this case, the diagnosis of left-sighted PJI was confirmed by prosthesis replacement. Figure 2 shows an example of inflammation of the periprosthetic bone marrow.

In the analysis of the MRI datasets alone, the periprosthetic bone margin could not be reliably assessed in eight prostheses and bone marrow in six patients because of severe artefacts. With simultaneous PET/MRI, all ten confirmed PJIs were correctly diagnosed or excluded (sensitivity, 100%; specificity, 100%). Table 3 shows the results of PET/MRI assessment according to the reference standard. Considering the anatomical areas, using PET/MRI, the periprosthetic bone margins could not be reliably assessed due to severe artefacts of the two prostheses. PET/MRI was false positive in the bone marrow area and periprosthetic bone margin in two and one protheses, respectively. False-negative findings in the periprosthetic bone margin and bone marrow were observed in three and one arthroplasties, respectively. The mean SUVmean and SUVmax in PJI were 3.6 ± 1.9 and 6.7 ± 3.7, 3.7 ± 2.1 and 6.7 ± 4.3, and 3.7 ± 1.8 and 6.9 ± 3.5 in the periprosthetic bone margin, bone marrow, and soft tissue, respectively.

**Table 3:** Diagnostic accuracy for the diagnosis of a periprosthetic joint infection by positron emission tomography/magnetic resonance imaging separated for the predefined anatomical areas. ^*^The analysis of the anatomical regions includes the results of the surgically obtained samples (10 each of the periprosthetic bone margin and soft tissues and 7 samples of the bone marrow)

Diagnostic accuracy of PET/MRI for the diagnosis of PJI	Anatomical region^*^			Overall hip or knee arthroplasty
	Periprosthetic bone margin	Bone marrow	Soft tissue	
Sensitivity (%)	57.1	75	100	100
Specificity (%)	50.0	33.3	100	100
PPV (%)	80.0	60.0	100	100
NPV (%)	25.0	50.0	100	100
True positive	4	3	9	10
True negative	1	1	1	3
False positive	1	2	0	0
False negative	3	1	0	0
Not evaluable	1	0	0	0

The pathogens found in cultures were *Corynebacterium mucifaciens* (1), *Staphylococcus capitis* (1), *Staphylococcus aureus* (1), *Staphylococcus epidermidis* (2), *Enterococcus faecalis* (2), *Cutibacterium acnes* (1), *S. epidermidis and S. capitis* (1), *Streptococcus* species, and *Corynebacterium* species (1).

## Discussion

This study demonstrates that PJI can be reliably diagnosed using simultaneous ^18^F-FDG-PET/MRI with a sensitivity and specificity of both 100%. The challenge of conservative imaging methods is the detection of tissue changes, which often can only be detected radiographically and by CT after a prolonged infection. Furthermore, post-operative MRI signs of PJI are controversially discussed in the literature.

The use of metal artefact-reducing MRI sequences greatly improves image quality and has been successfully used to assess complications after joint replacement [21, 22]. Previous studies have shown in a collective of 140 cases on a 1.5 T MRI scanner that the presence of periosteal reactions, capsular oedema, and intramuscular oedema after total hip arthroplasty has high accuracy in evaluating PJI, resulting in sensitivity and specificity of 78–95% and 86–95%, respectively, for the diagnosis of PJI [23]. Periosteal bone formation was significantly specific (100%), but with low sensitivity (16%) [24]. Although intramuscular collections are specific for PJI, they are not always present. PET/MRI showed that the highest sensitivity and specificity (100% and 100%) were achieved in the evaluation of the anatomical regions when assessing the soft tissues. MRI assessment of the periprosthetic bone margin was not possible in eight patients because of severe artefacts. We used a hybrid simultaneous PET/MRI scanner with a field strength of 3.0 T. Recent MRI studies have shown that metal artefacts are lower when scanning at a field strength of 1.5 T than at a field strength of 3.0 T [22]. This could be a reason for the reduced MRI assessment of the periprosthetic bone margin and bone marrow in our study. Ultimately, the use of artefact-reducing sequences allowed for a significantly good anatomic mapping of the tracer uptake of the PET data. With the addition of PET studies, the periprosthetic bone margin could not be assessed in two patients due to severe artefacts.

In recent years, nuclear medicine techniques for the diagnosis of PJI have been well studied. Hence, ^18^F-FDG PET has shown great promise for the evaluation of infections and inflammation in several studies [14]. However, in the diagnosis of PJI, there are different results regarding sensitivity and specificity. In Verbene et al.’s meta-analysis, the sensitivity and specificity of ^18^F-FDG-PET were 70% and 84%, respectively, and ^18^F-FDG-PET was less specific in diagnosing periprosthetic knee infection than combined leukocyte and bone marrow scintigraphy and antigranulocyte scintigraphy [12]. Another meta-analysis reported that the pooled sensitivity and specificity of ^18^F-FDG-PET or PET/CT in detecting PJI were 86% and 86%, respectively [25], which were lower than those in our study. Although the detection of patients with an overall diagnosis of PJI by PET/MRI was significantly reliable, there were six misdiagnoses in the periprosthetic bone and adjacent marrow area on anatomical review. An important reason for these results could be the lack of uniform criteria for the interpretation of PET findings [14]. Nonspecific ^18^F-FDG accumulation around the head and neck area of the prosthesis may be present post-operatively for a long time over several months after implantation. Currently, the location and pattern of FDG accumulation appear to be more important than the uptake intensity at these sites [26–28]. This finding was published in 2002 by Chacko et al. in patients with hip arthroplasty [28]. The authors concluded that the intensity of increased FDG uptake is less important than the location of increased FDG uptake when FDG-PET is used to diagnose PJI and that using an increased tracer uptake as the sole criterion for diagnosing PJI, a higher rate of false-positive results will occur. Chacko et al. emphasised in their study that the images from patients with histologically proven PJI displayed increased tracer uptake along the interface between the bone and prosthesis and that the intensity of the increased tracer uptake varied from mild to moderate, with SUV less than 2. In contrast, images from uninfected, loose hip prostheses revealed significantly intense focal uptake around the head or neck of the prosthesis with SUV as high as 7. Other investigators have also shown that quantitative SUV measurements cannot reliably differentiate between infection and aseptic loosening [29]. Therefore, we did not perform SUV-based diagnosis and focused on the pattern of ^18^F-FDG-accumulationn. According to our inclusion criteria without patients with evident PJI, for example, joint empyema, mild to moderate tracer uptake corresponds with the corresponding lower SUV values, which also support the above-mentioned results of Chacko et al.

In contrast, the different results could be due to the different examination techniques. Occasionally, ^18^F-FDG-PET examinations were performed with a stand-alone PET system, whereas others performed integrated PET/CT scan, which has become the standard method at most institutions. Metallic endoprostheses cause strong streak artefacts in CT images, which may lead to over- or underestimation of periprosthetic activity concentration and hamper semiquantitative PET evaluation when CT-based attenuation correction of PET images is used. This may explain the discrepancy in diagnostic performance proportionally. However, the anatomic discriminative resolution of PET/CT is lower than that of MRI. PET/MRI hybrid imaging potentially detects changes in the infected tissue with the highest contrast and spatial resolution of periprosthetic bone and soft tissue [15, 30]. Ultimately, artefact susceptibility remains a problem and is highly dependent on the prosthesis type. Even with the addition of PET data, the periprosthetic bone margin could not be reliably assessed in two patients in our study due to severe artefacts. Nevertheless, an important advantage of PET examination is the imaging of the whole body and the detection of possible further foci of infection that may require treatment [31]. The impact of nuclear medicine or hybrid imaging techniques in the evaluation of infections and inflammations is becoming more and more important worldwide. However, there are no studies on PET/MRI in the field of PJI [32, 33].

This study has some limitations. A perfect test would have a sensitivity of 100% and a specificity of 100%. We also obtained these results, but our evaluation is based on a small number of patients, heterogeneous patient population, and limited statistical power. One of the most common causes of PJI is haematogenous spread [34]. Therefore, in patients with suspected PJI, we considered all implanted arthroplasties as potentially infected and included them in the study. Finally, there was no justifiable indication for surgical specimen collection in the three non-painful arthroplasties, so only the result of a puncture with microbiological analysis was evaluated. We did not perform a separate evaluation of the MRI and PET datasets because the MRI data evaluation was limited. In fact, 1.5 T scanners are better suited for examining prostheses and implants. Microbiological evaluation of our cohort revealed that both high-and low-virulence pathogens were detected. Although the number of patients was small for significant statistical analysis, no trend in FDG uptake was observed. This should be verified in a large-scale study.

## Conclusion

Simultaneous ^18^F-FDG-PET/MRI imaging provides a good modality for assessing PJI both functionally and anatomically and reliably in soft tissues. Especially for the functional information derived by ^18^F-FDG-PET, the pattern of FDG accumulation is more important for diagnosing PJI than the intensity of tracer uptake derived by quantitative SUV measurement. With combined PET/MRI, there were no false-negative results, indicating that combined ^18^F-FDG PET/MRI may be useful for the exclusion of PJI in patients with persistent painful prostheses and clinically suspected PJI. The hypothesis that PET/MRI provides reliable results of the extent of infection of the individual regions is to be viewed critically in the periprosthetic bone region and bone marrow. However, it remains to be determined whether this gain justifies the high time and cost involved. Further technical improvements in artefact removal and measurement time reduction could be useful for future re-evaluation to assess efficient use in clinical practice.

## Supplementary Information

Below is the link to the electronic supplementary material.

Supplementary file1 (DOCX 19 kb)

## Data Availability

All relevant data generated or analysed during the current study are presented in this paper.
