# Supplementary material for: Simultaneous 18F-FDG-PET/MRI for the detection of periprosthetic joint infections after knee or hip arthroplasty: a prospective feasibility study
Source: Int Orthop. 2022 May 30;46(9):1921–8. doi: 10.1007/s00264-022-05445-7 (PMC9372014; doi:10.1007/s00264-022-05445-7)
Supplement: Supplementary file 1 — Supplementary file1 (DOCX 19 kb) [file 264_2022_5445_MOESM1_ESM.docx]

**Supplemental table 1**

| No. | Prosthesis localisation | | Image Findings in PET/MRI | Pathogen detection |
| --- | --- | --- | --- | --- |
| 1 | Hip | Right | Edema in the muscle and soft tissue  Increased FDG uptake in the soft tissue, , around the neck and shaft of the prosthesis | *Corynebacterium mucifaciens* |
| 2 | Hip | Right | Joint effusion with CA enhancement, edema in the muscle and soft tissue, muscle atrophy  Increased FDG uptake in the soft tissue around the neck of the prosthesis | *Staphylococcus capitis, Staphylococcus epidermidis* |
| 3 | Hip | Left | Discrete edema in the soft tissue  No increased FDG uptake | *none* |
| 4 | Hip | Left | Fluid collections and edema in the soft tissue, muscle edema  Increased FDG uptake in the soft tissue | *Staphylococcus capitis* |
| 5 | Hip | Right | Fluid collections and edema in the soft tissue, muscle edema  Increased FDG uptake in the soft tissue and the periprothestic bone margin in the acetabulum | *Staphylococcus aureus* |
| 6 | Hip | Left | Edema in the muscle and soft tissue  Increased FDG uptake in the soft tissue | *Enterococcus faecalis* |
| 7 | Knee | Right | Fluid collections and edema in the soft tissue, joint effusion  Increased FDG uptake in the soft tissue and distal bone marrow | *Enterococcus faecalis* |
| 8 | Knee | Left | Edema in the muscle and soft tissue  Increased FDG uptake in the soft tissue and the periprothestic bone margin in the femur and tibia | *Staphylococcus epidermidis* |
| 9 | Knee | Right | Joint effusion, fluid collections with CA enhancement and edema in the soft tissue,  Increased FDG uptake in the soft tissue and distal bone marrow | *Staphylococcus epidermidis* |
| 10 | Knee | Left | Discrete edema in the soft tissue  No increased FDG uptake | *none* |
| 11 | Hip | Right | Joint effusion, edema in the muscle and soft tissue with fluid collections with CA enhancement  Increased FDG uptake in the soft tissue and distal bone marrow, around the neck and shaft of the prosthesis | *Cutibacterium acnes* |
| 12 | Hip | Right | Discrete edema and some fluid collection in the soft tissue  No increased FDG uptake | *none* |
| 13 | Hip | Left | Edema in the muscle and soft tissue, fluid collections with CA enhancement  Increased FDG uptake in the soft tissue, distal bone marrow, around the neck of the prosthesis | *Streptococcus species, Corynebacterium species* |

Supplemental table 1: Image findings in ^18^F-FDG-PET/MRI and pathogen detection. ST soft tissue, CA contrast agent.
